# Role of the Pruritic Cytokine IL-31 in Autoimmune Skin Diseases

**DOI:** 10.3389/fimmu.2019.01383

**Published:** 2019-06-21

**Authors:** Bernhard F. Gibbs, Nikolaos Patsinakidis, Ulrike Raap

**Affiliations:** Division of Experimental Allergy and Immunodermatology, University of Oldenburg, Oldenburg, Germany

**Keywords:** IL-31, itch, autoimmunity, bullous pemphigoid, psoriasis, eosinophils, basophils

## Abstract

Many autoimmune skin diseases, such as bullous pemphigoid (BP), psoriasis and certain types of chronic urticaria, are associated with intensive pruritus. While histamine and neuropeptides have previously been ascribed to play a role in itch that accompanies these diseases, recent evidence suggests that the pruritogenic cytokine interleukin (IL)-31 is a major driver of pruritic responses. IL-31 was originally shown to be produced by activated helper T cells, particularly Th2 cells, mast cells, macrophages and dendritic cells. However, more recent evidence demonstrated that eosinophils are a major source of this cytokine too, particularly in bullous pemphigoid. Basophils have also been shown to express the cytokine which, through autocrine action, strongly supports the production of other Th2-type cytokines from these cells. These investigations suggest that the dynamic recruitment of eosinophils and basophils in some autoimmune skin diseases could play an important role in the severity of IL-31-mediated itch. Furthermore, these studies suggest that IL-31, in addition to its pruritic actions, also has potential immunomodulatory roles in terms of supporting Th2-type immunity, which often underpins IgE-associated autoimmune diseases (such as bullous pemphigoid and urticaria) as well as allergies. While the role of IL-31 in psoriasis remains to be clarified, current evidence shows that this cytokine plays a major role in BP, chronic spontaneous urticaria and dermatomyositis. This suggests potential use of IL-31 receptor-blocking therapeutic approaches (e.g., Nemolizumab) for the treatment of IL-31-associated disorders.

## Introduction

IL-31 was first described by Dillon et al as a T cell-derived cytokine that was predominantly produced by The cells (1). Interestingly, mice which overexpressed IL-31 were observed to develop severe pruritus, alopecia and skin lesions (1). Furthermore, animal models of airway hypersensitivity exhibited increased IL-31 receptor expressions and these expressions, as well as those of IL-31 receptors in the skin, were also verified in respective human tissues (1). Human and mouse IL-31 share 31% amino acid homology and the genes for this cytokine are, respectively, located on chromosome 12q24.31 and chromosome 5 (1).

IL-31 is a member of the gp130/IL-6 family of cytokines and is a four helix bundle cytokine that acts as a ligand for the heterodimeric IL-31 receptor A (IL-31RA), a novel type I cytokine receptor, and the oncostatin M receptor (OSMR), the latter of which increases IL-31 binding affinity to IL-31RA. Both of these IL-31 receptors are expressed on a variety of different cell types including T cells, dorsal root ganglia (DRG), keratinocytes, dendritic cells, eosinophils, basophils, and macrophages (2–7). The functional activity of IL-31 appears to depend on the expression of both the IL-31RA and OSMR since, in basophils, we recently demonstrated that blocking of either receptor type decreases IL-31-induced IL-4 and IL-13 release (7).

Subsequent signaling following receptor complex binding involves the Janus kinase–signal transducer and activator of transcription pathway, the phosphoinositide-3-kinase–Akt pathway as well as the mitogen-activated protein kinase pathway (4, 8–10). IL-31 signaling controls a wide range of biological functions and immunomodulatory effects, such as the release of chemokines, proinflammatory cytokines, regulation of cell proliferation and stimulation of DRG sensory neurons which are responsible for induction of itch [reviewed in (11, 12)].

## General Properties of IL-31 in Skin Diseases

In dermatological diseases, IL-31 is of particular interest in terms of itch and inflammation as well as impaired skin-barrier function arising from IL-31-induced tissue remodeling (13, 14). The cytokine was not only found to induce severe pruritus in mice, where levels of IL-31 correlated with scratching behavior, but a NC/Nga mouse model of atopic dermatitis demonstrated the potential therapeutic benefit of blocking anti-IL-31 antibodies (1, 15, 16). Before the discovery of IL-31, histamine was widely considered to be the most important pruritic mediator and the relative lack of therapeutic control of itch by H1-antihistamines was ascribed following the discovery of additional H4-receptor input in pruritus [reviewed in (17)]. This is partly supported by a recent successful clinical trial using an H4-receptor antagonist in atopic dermatitis (18), indicating potential future combinational H1- and H4-receptor antagonist/inverse agonist approaches. However, recent data from human clinical trials using nemolizumab (CIM331), a humanized antibody against interleukin-31 receptor A, clearly indicates that IL-31 is an important mediator of itch in humans too (19, 20).

Alongside its pruritogenic actions, IL-31 has a proinflammatory role too due to the upregulation of proinflammatory gene expressions in T cells, including CCL2 and granulocyte colony-stimulating factor (21). Interestingly, CCL2 has recently been implicated in driving basophil trafficking in systemic lupus erythematosus (SLE) and in severe allergic reactions (22, 23). IL-31 release by CD4+ T cells is mostly restricted to Th2 cells, although Th1 cells can produce this cytokine *in vitro* following exposure to IL-4 (21). Epidermal keratinocytes are also a major target for IL-31, where it induces chemokine gene expressions for GRO1α (CXCL1), I-309 (CCL1), MIP-1β (CCL4), TARC (CCL17), MIP-3β (CCL19), MDC (CCL22), and MIP-3 (CCL23), [(1), reviewed in(11)]. Furthermore, Singh et al. showed that IL-31 increased epidermal basal-cell proliferation in mice resulting in thickening of the epidermal skin layer but increased transepidermal water loss (14). This impairment of skin barrier function occurs due to IL-31 decreasing filaggrin expression in human keratinocytes (24). Interestingly, reduced filaggrin expressions are strongly associated with atopic dermatitis and have been shown to enhance *Staphylococcus aureus* colonization (24, 25).

Increased levels of IL-31 have now been shown in various inflammatory skin diseases including prurigo nodularis (12), atopic dermatitis (26–28), contact eczema (29), chronic spontaneous urticaria (CsU) (30), as well as in a subset of patients with mastocytosis (31). In an animal model of atopic dermatitis anti-IL-31 treatment significantly reduced scratching in mice (16), underlining the important role of IL-31 in mediating pruritus. This seems to apply to humans too, where IL-31 levels were shown to correlate with disease severity in atopic dermatitis (27, 28). Interestingly, CsU patients respond to omalizumab therapy which was recently shown to be associated with reduced IL-31 serum levels (32). This suggests that this IL-31 may be modulated by IgE-dependent mechanisms and this further highlights the potential of anti-IL-31 as a therapeutic approaches as well as targeting IgE-positive effector cells.

## IL-31 in Autoimmune Skin Diseases

IL-31 has thus far been shown to play a prominent role in common inflammatory skin diseases, such as atopic dermatitis, particularly in relation to pruritus. However, since pruritus is also a major feature in bullous pemphigoid, chronic spontaneous urticaria (CsU) and other autoimmune diseases there is a potential role for IL-31 in some of these diseases too.

### Chronic Spontaneous Urticaria

It has long been recognized that autoimmune mechanisms are an important component of CsU [reviewed in (33)]. CsU is defined by the rapid appearance of pruritic wheals lasting <24 h but which may occur repeatedly over a period of at least 6 weeks. The autoimmune association in CsU is either due to IgE mediated autoallergy against thyroid peroxidase or IgG auto-antibodies against the α-subunit of the high-affinity IgE receptor(FcεRI) and, more rarely, against IgE. All of these possible autoimmune mechanisms activate mast cells and basophils, cells which have been centrally implicated with CsU (34–36).

While mast cells and basophils are the main sources of histamine, antihistamine treatment in patients with CsU does not fully eliminate itch, suggesting that IL-31 may have some input in pruritic mechanisms associated with CsU. Indeed, we observed increased IL-31 serum levels in CsU patients, although these levels were generally lower than those seen in atopic dermatitis patients (30). These observations have recently been confirmed by Lin et al. who demonstrated significantly higher levels of IL-31 in CsU patients with most severe pruritus intensity compared to milder forms but not with urticarial activity *per se* (37). This supports the notion that IL-31 contributes to itch rather than other aspects of disease activity.

In our own investigations, we subsequently discovered that the main cellular sources of IL-31 in skin lesions of CsU patients are basophils (7). Basophils produced and released IL-31 in response to IgE-dependent stimulation and were even more responsive in terms of secreting the cytokine following incubation with the bacterial peptide *N*-Formylmethionyl-leucyl-phenylalanine (7). Basophils also expressed both IL-31 and OSMR receptors, and were responsive to IL-31 stimulation which gave rise to release of the archetypal Th2-type cytokines IL-4 and IL-13 from these cells. Surprisingly, however, IL-31 failed to cause degranulation of basophils, as determined by the expression of the degranulation markers CD63 and CD203c as well as histamine release (7).

Since basophils are predominant sources of both IL-31 and histamine, inhibition of basophil function is of therapeutic interest in controlling itch-related symptoms in CsU. Furthermore, basophils not only significantly infiltrate the skin in CsU (38) but are also crucial contributors of IL-4 and IL-13 which support IgE class-switching and, in the case of IL-4, also Th2 immunity [reviewed in (39)]. IgE-dependent basophil activation can be therapeutically targeted with the monoclonal antibody omalizumab, which prevents free-circulating IgE from binding to cells such as basophils and also mast cells, leading to the down-regulation of cell-surface FcεRI expressions as well as the degree of IgE sensitization. Since the autoimmune component of CsU is thought to involve autoantibodies against either FcεRI or IgE a reduction of cell-surface IgE and/or FcεRI due to omalizumab presents an important therapeutic strategy. Indeed, omalizumab has been used successfully to treat patients with CsU (40, 41). The fact that IL-31 levels are also reduced following omalizumab treatment further supports the role of basophils in CsU (32). This is underlined by reports of a rapid reduction *in vivo* of both FcεRI and IgE expressions on basophils due to omalizumab (42). While mast cells may also be targeted by omalizumab, the reductions in FcεRI receptor density resulting from omalizumab therapy are known to occur sooner in basophils than for skin mast cells, possibly due to a greater turnover of basophils compared to their skin tissue-fixed mast cell counterparts (43).

In addition to our observations regarding IL-31 inducing Th2-type cytokine release from basophils, we found that the cytokine also facilitates basophil chemotaxis (7). This indicates that IL-31 plays a role in the orchestration and accumulation of basophils in inflammatory skin diseases such as CsU. Since basophils are majorly involved in the pathogenesis of CsU, targeting IL-31 directly (e.g., by nemolizumab) may prevent both IL-31-mediated basophil recruitment as well as the autocrine effects of this cytokine in stimulating the release of IL-4 and IL-13 from basophils. Such approaches using nemolizumab (CIM331) have recently been tested successfully (also in terms of reducing itch) in atopic dermatitis in both humans and other primates (19, 20, 44–46). Similarly, the anti-canine-IL-31 monoclonal antibody, lokivetmab, has been used to treat atopic dermatitis in dogs (47–49). However, the potential clinical benefits of anti-IL-31 monoclonal antibodies have yet to be reported for CsU (and indeed other autoimmune skin diseases).

### Bullous Pemphigoid

The autoimmune blistering skin disease, bullous pemphigoid (BP), is accompanied by severe pruritus, thus suggesting possible involvement of IL-31. Indeed, Salz et al reported high IL-31 levels in BP blister fluids as well as an association of this cytokine with granulocytes (50). More recently, we also showed that IL-31 is expressed in the lesional skin of BP patients, particularly in eosinophils from skin tissue and also within blister fluids (51). Moreover, eosinophils were demonstrated to be the major cellular source of IL-31 in BP and isolated peripheral blood eosinophils secreted substantial amounts of IL-31 (51). Similarly to basophils, IL-31 also has chemotactic effects on eosinophils and additionally stimulates these cells to release reactive oxygen species and the chemokine CCL26 (5).

It is, however, as yet unclear whether BP patients display elevated serum levels of IL-31, since the currently published literature has reported conflicting observations. Our own investigation showed only minor elevations in serum IL-31 levels vs. non-BP controls (51) compared to Salz et al (50), who observed significant increases, and Kulczycka-Siennicka et al. (52), who reported reduced levels of the cytokine. Despite these different observations regarding IL-31 serum levels, it is clear that eosinophils are one of the major sources for the increased IL-31 levels in BP blister fluids, which often contain a highly enriched accumulation of eosinophils. Because BP patients experience pruritus particularly in lesional skin the increases in IL-31 levels in blister fluids offer a plausible explanation for this localized itch.

Basophils have also been reported to be associated with BP (53) but their potential contribution to IL-31 in this disease is not yet known. Eosinophil-derived IL-31 may possibly participate in the trafficking of basophils to lesional tissues affected by BP and this may further be supported by IL-31-induced CCL26 release from eosinophils (5). Furthermore, there are several case reports of successful therapy of BP using omalizumab, suggesting potential basophil involvement (54–56). However, Freire et al recently showed that the mast cells and eosinophils make up most of the IgE-expressing cells in BP skin (57) and both cell types are widely reported to participate in various autoimmune skin diseases [reviewed in (58, 59)]. It is thus conceivable that the clinical benefit of omalizumab therapy may largely due to a reduction in eosinophil and mast cell function.

### Psoriasis

It was originally thought that IL-31 does not play a role in psoriasis, based on comparisons of cellular expressions of the cytokine (both at mRNA level and immunoreactivity) together with controls (12, 29, 60). However, more recent investigations have reported that serum IL-31 levels are significantly elevated in psoriasis and that chronic itch associated with psoriatic skin is accompanied by increased transcription of IL-31 (61, 62). Narbutt et al further showed that IL-31 serum levels were significantly reduced after narrowband UVB phototherapy, coinciding with a substantial reduction in pruritus in these patients (61). This study indicates that itch accompanying psoriasis might be associated with IL-31. Interestingly, skin mast cells have been shown to express elevated IL-31 in psoriatic skin compared to healthy controls (63). In contrast, however, Czarnecka-Operacz et al. failed to observe any correlation between itch severity in psoriasis and IL-31 (64). On balance, and despite the possibility that it may contribute to pruritic forms of the disease, the currently published literature does not yet provide a clear consensus regarding the role of IL-31 in psoriasis *per se*.

### Autoimmune Connective Tissue Diseases Affecting the Skin

Pruritus is a prominent symptom in several autoimmune connective tissue diseases such as lupus and dermatomyositis, although these diseases can affect multiple organ systems and are often supervised by dermatologists. In lupus erythematosus there is currently only sparse evidence to suggest a role of IL-31 and, as yet, no reports specifically dealing with cutaneous forms of this autoimmune disease. Although pruritus does often accompany cutaneous lupus erythematosus it is usually mild in severity and (65), possibly indicating that IL-31 is not a major player in this disease. In systemic lupus erythematosus (SLE) IL-31 serum levels were not shown to be significantly increased compared to healthy controls (66). However, in stark contrast, a more recent study reported significant increases in IL-31 serum levels in SLE as well as identification of IL-31 polymorphisms that were associated with SLE in the Chinese population (67). While the role of IL-31 in SLE is still unclear these IL-31 polymorphisms may nonetheless serve as novel genetic markers of susceptibility to SLE (67).

Dermatomyositis is another autoimmune disease that involves the skin and is sometimes involves considerable pruritus(68), although the muscles and sometimes the lungs can also be affected. In a recent article, Kim et al showed that itch correlated with increased cutaneous severity where IL-31 and IL-31RA gene expressions in lesional skin were upregulated compared with either non-lesional skin or that from healthy controls (69). IL-31 mRNA expression also positively correlated with itch score and immunoreactivity for IL-31 and IL-31RA was greater in lesional skin. Furthermore, lesional dermatomyositis skin contained significantly more IL-31-producing cells, of which CD4+ cells were the most abundant IL-31-expressing cell type (70).

### Alopecia Areata

In addition to its role in pruritic skin diseases, IL-31 was originally also associated with alopecia areata, an autoimmune disease involving inflammation of the hair follicles and a loss of immune privilege [reviewed in (70)]. However, although IL-31 and hair loss was described in mice (1) it was not subsequently observed in patients with alopecia areata (60).

## Concluding Remarks

**Figure 1:**
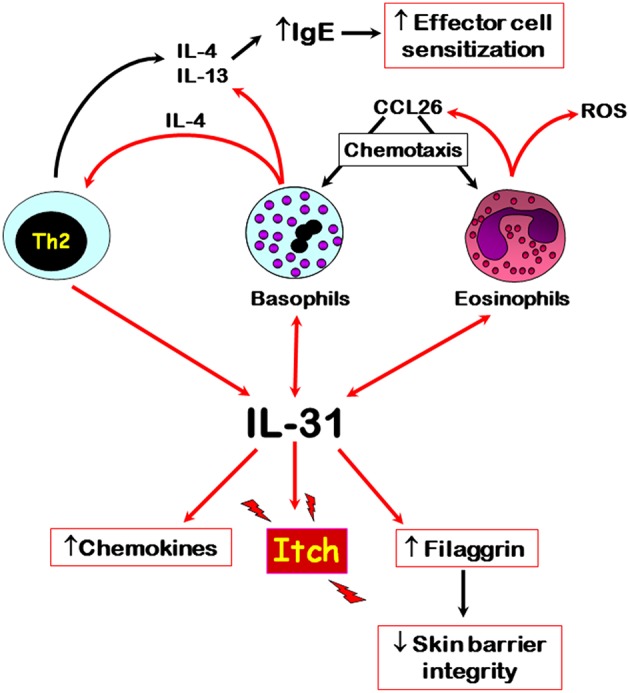
Overview of the main cellular sources and potential roles of IL-31 in autoimmune skin diseases. Red arrows denote direct effects, black indirect effects. Double arrows denote autocrine activation.

The published literature to date shows that IL-31 is differentially associated with autoimmune skin diseases, especially those where pruritus is a major symptom, such as CsU and bullous pemphigoid. Here, recent evidence shows that basophils and eosinophils are major sources of this cytokine, respectively, especially within affected skin tissues. However, the input of other IL-31-producing cells, especially CD4+ T cells, in these autoimmune diseases still needs to be elucidated in terms of IL-31-mediated immunomodulatory roles. IL-31 possibly plays a role in the trafficking of basophils to affected tissue sites because of its known actions in supporting the release CCL2 and CCL26 chemokines. Basophil-derived IL-4, the release of which is also driven by IL-31, may support the development of Th2 cells and subsequent further IL-31 production. The interplay of IL-31 and various immune cells that are known to play a role in autoimmune skin diseases is summarized in Figure 1. Overall, IL-31 is a crucial driver for pruritus in certain autoimmune skin diseases and the recent development of blocking antibodies offers exciting new therapeutic opportunities to combat itch-related symptoms.

## Author Contributions

All authors listed have made a substantial, direct and intellectual contribution to the work, and approved it for publication.

### Conflict of Interest Statement

The authors declare that the research was conducted in the absence of any commercial or financial relationships that could be construed as a potential conflict of interest.
